# Genomic language models could transform medicine but not yet

**DOI:** 10.1038/s41746-025-01603-4

**Published:** 2025-04-18

**Authors:** Micaela Elisa Consens, Ben Li, Anna R. Poetsch, Stephen Gilbert

**Affiliations:** 1 Department of Computer Science, University of Toronto, Toronto, ON Canada; 2 Vector Institute for Artificial Intelligence, Toronto, ON Canada; 3 Peter Munk Cardiac Center, University Health Network, Toronto, ON Canada; 4 Division of Vascular Surgery, University of Toronto, Toronto, ON Canada; 5 Temerty Centre for Artificial Intelligence Research and Education in Medicine, University of Toronto, Toronto, ON Canada; 6 Biomedical Genomics, Biotechnology Center, Center for Molecular and Cellular Bioengineering, Technische Universität, Dresden, Germany; 7 National Center for Tumor Diseases (NCT) partner site Dresden, German Cancer Research Center (DKFZ), Dresden, Germany; 8 Carl Gustav Carus University Hospital Dresden, Dresden University of Technology, Dresden, Germany; 9 Else Kröner Fresenius Center for Digital Health, TUD Dresden University of Technology, Dresden, Germany

**Keywords:** Health care, Health policy

## Abstract

Recently, a genomic language model (gLM) with 40 billion parameters known as Evo2 has reached the same scale as the most powerful text large language models (LLMs). gLMs have been emerging as powerful tools to decode DNA sequences over the last five years. This article examines the emergence of gLMs and highlights Evo2 as a milestone in genomic language modeling, assessing both the scientific promise of gLMs and the practical challenges facing their implementation in medicine.

## Introduction

In February 2025, researchers announced Evo2, a genome language model (gLM) trained on over 128,000 genomes, encompassing over 9.3 trillion DNA base pairs^1^. This computational scale matches leading text-based LLMs, representing a significant milestone for genomic AI^2^. Unlike protein language models, which train to understand the 2% of human DNA that is encoded into amino acids and folded into proteins, gLMs train to understand the entire genome^3^. This largely consists of understanding the role of the remaining 98% of human DNA that is non-coding. Non-coding DNA contains crucial regulatory elements that coordinate gene expression across different cell types and developmental stages^4^, and the precise mechanisms governing this regulation are increasingly being unraveled. This field of study is known as regulatory genomics^4^, and gLMs have emerged as promising tools to study it. The introduction of Evo2 represents both important progress for the field and highlights critical questions about what these models learn and how they might be applied. This article examines gLMs in the context of Evo2, highlighting their potential for biological research and medicine while exploring the technical barriers and ethical challenges—from data privacy to dual-use risks—that will shape their clinical future.

## Training of gLMs

Pre-training is an initial learning phase, where gLMs are trained on large amounts of DNA sequence data, to learn the underlying patterns and grammar of the genome. Just as human language grammar provides rules for constructing meaningful sentences, genomic grammar consists of patterns and rules that govern how DNA sequences are shaped by evolution. gLM pre-training is typically self-supervised, meaning it is done on data without labels, and usually as a reconstruction task. A reconstruction task requires the model to learn to “fill in” missing parts of the input data, where success is measured by how accurately the model reconstructs the original sequence. The Evo2 model trains to predict the next nucleotide in a genomic sequence, the same way LLMs train to predict the next words in a sentence. To reconstruct missing genomic data, gLMs like Evo2 compress genomic information into learned representations that potentially capture the semantic information within DNA sequences. Once learned during pre-training, these representations can be leveraged during a second phase of training known as fine-tuning. Fine-tuning is typically done on smaller, well-curated, and labeled datasets for specific biologically relevant tasks like predicting regulatory elements (regions involved in coordinating gene expression), segmenting genomic regions (locating the boundaries of functional regulatory elements), and more^5^. This is a departure from conventional genomic machine learning approaches, which have traditionally relied on supervised learning with task-specific labeled datasets (such as experimental assay data), whereas gLMs aim to learn universal genomic representations that can be adapted across multiple tasks through finetuning^5^.

The current paradigm for training gLMs involves unsupervised pre-training on as many diverse species’ genomes as possible, since the functional importance of DNA sequences for genes and gene regulation is conserved across evolution^6,7^. Evo2 dramatically extends this approach by training on over 128,000 genomes, compared to the previous largest model that trained on 850 genomes^6^. This evolutionary conservation helps provide recurrent signals from conserved sequence amidst noise from non-conserved sequence, as researchers still debate how much of the non-conserved genome contributes to gene regulation^8^. Large sections of the genome contain long repetitive sequences with unknown functional significance for gene regulation. Recent gLMs, including Evo2, increase the focus on sequences relevant for gene regulation by employing weighted loss schemes that reduce the contribution of repetitive elements during training, which improves overall performance for related tasks^9^.

Another trend in gLM modeling has been increasing model context size, which is the length of DNA sequences a model can ‘see’ at once. This is an effort to model long-range interactions in the genome, and potentially even model the entire human genome at once. Evo2 specifically adopts an architecture that radically increases its context size compared to most gLMs; handling sequences up to 1 million nucleotides long. While an impressive advancement, this still falls short of the context required for whole human chromosomes, which can span hundreds of millions of nucleotides. Moreover, there exists a trade-off between context length and interpretability; Evo2’s complex architecture enables its large context window but makes the model more difficult to interpret compared to simpler models with shorter contexts (Table 1).

**Table 1:** Comparison of recent gLMs with multi-species and single-species training approaches

Model	Parameters	Sequence length (in bp)	Genomes trained on	Human genome included	Training type
GPN MSA^9^	86,000,000	128	100	Yes	Multi-species
GPN^20^	65,612,800^*^	512	8	No	Multi-species
Evo2^1^	40,000,000,000	1,000,000	128,000	Yes	Multi-species
Nucleotide transformer^6^	2,500,000,000	6000	850	Yes	Multi-species
DNABERT-2^21^	117,000,000	877 (BPE)	135	Yes	Multi-species
DNABERT^13^ (*k* = 6)	110,000,000	512	1	Yes	Single-genome
HyenaDNA^22^	1,600,000	1,000,000	1	Yes	Single-genome
GROVER^15^	86,511,201^*^	2076 (BPE)	1	Yes	Single-genome

This table compares eight gLMs based on their number of parameters, training data composition, and sequence handling capabilities. Parameter numbers are taken from the paper where possible, where indicated by^*^, the model's parameter number is calculated from loading the HuggingFace model version. Models are categorized by their training approach (multi-species vs single-genome). For sequence length calculation, measurements are in DNA base pairs (bp). When tokens represent multiple bp, the total input length was calculated by multiplying tokens by bp per token (e.g., nucleotide transformer uses non-overlapping *k*-mers where *k* = 6, so 1000 tokens = 6000 bp). DNABERT-2 and GROVER use Byte Pair Encoding (BPE), which has varying length tokens based on the co-occurrence frequency of the characters and a pre-defined vocabulary size. Note that DNABERT-2’s sequence length estimation (877) represents approximately 128 tokens at 6.85 bp average per BPE token (calculated from HuggingFace https://huggingface.co/zhihan1996/DNABERT-2-117M/blob/main/tokenizer.json vocabulary excluding special tokens), and GROVER’s sequence length (2076) represents approximately 510 tokens at 4.07 bp average per BPE token.

## The biological and clinical relevance of gLMs

Pre-training gLMs has immense potential for biology through what researchers call ‘zero-shot’ performance—a model’s ability to perform well on tasks it wasn’t explicitly trained for. Strong zero-shot performance indicates the model has learned fundamental principles about genomic structure that generalize to new scenarios. When a gLM pretrains in a self-supervised manner, it enhances its ability to uncover novel biology independent of pre-existing human annotations and expectations. Potentially, this means gLMs with strong self-supervised zero-shot performance have uncovered new regulatory grammar within the genome—grammar that we can learn from. Uncovering novel genomic grammar would advance our understanding of human disease and transform personalized care across all aspects of medicine. Given that almost all the leading causes of death/disability in the world have an important genetic component^10^, it is likely that in the future, gLMs could help clinicians estimate the risks of whether a patient will develop these diseases, years before their onset, and implement appropriate personalized preventive strategies.

## Challenges and opportunities in the clinical adoption of gLMs

Despite Evo2’s impressive scale and capabilities, fundamental questions remain about what these models are learning. A critical challenge is determining whether gLMs learn contextual relationships within genomic sequences or simply memorize patterns from training. This evaluation challenge is compounded by two factors: the reliance on simple benchmarks for evaluation and the multi-species training approach. While training on diverse species helps Evo2 and similar models identify functionally important sequences, it also makes it difficult to distinguish between true understanding and recall of evolutionarily similar sequences at prediction time.

### Understanding vs memorization

Many gLMs report success on simple benchmarking tasks that fail to capture the complexity of genomic regulation^11,12^. These benchmarks, such as distinguishing real genomic sequences from randomly generated ones, are used primarily because they’re computationally tractable and provide clear evaluation metrics, but they do not reflect the true challenges of interpreting regulatory grammar and frequently are driven by DNA sequence motifs which can be learned without the need to grasp larger context. Designing biologically meaningful benchmarks is challenging, as ground truth labels are often only available in small datasets insufficient for model training. Consequently, researchers generate datasets from less well-validated data and provide synthetic random sequences as controls to avoid introducing confounding genomic signals. However, this approach often fails to test models on the complex regulatory patterns they are ultimately intended to discover.

Research on earlier gLMs like DNABERT^13^ revealed they primarily learned sequence patterns through recalling training data rather than understanding deeper contextual relationships^14^. Similarly, the GROVER model, recently described by Sanabria and colleagues (2024), demonstrated that gLMs initially learn token frequencies^15^, which may inhibit their ability to capture complex contextual relationships in genomic data. Sanabria and colleagues^15^ additionally showed that even a simple model focusing solely on token frequencies performs well on many benchmarking tasks, supporting the idea that current evaluation methods lack robustness.

### Generation capabilities and their limitations

Evaluation challenges extend beyond fine-tuned tasks to the pre-trained capabilities of gLMs. Evo2 is pretrained on next nucleotide prediction, which enables it to generate novel genomic sequences without further training. With its impressive 1 million base pair context window, Evo2 can theoretically generate entire prokaryotic and simple eukaryotic genomes^1^. As of now, generation-evaluation primarily measures the statistical properties of generated sequences as compared to real genomes using bioinformatic tools, rather than assessing their biological viability or function. Importantly, none of Evo2’s generated genomes have been synthesized in a laboratory and tested for viability in living cells. Furthermore, many evaluations of Evo2’s generation capabilities resemble recall tests that potentially measure the model’s ability to reproduce sequences that are evolutionarily similar to those in its massive training dataset, rather than demonstrating genuine understanding of genomic grammar.

While these evaluation challenges raise questions about current model capabilities, they don’t diminish gLM's potential. Ultimately, Evo2 and other gLMs’ generation capabilities are likely to be adopted by biologists first for research purposes before transitioning to clinical applications. This is partly because these generated sequences require more rigorous evaluation, but also because they offer valuable opportunities to explore DNA beyond known sequences. Synthetic sequences provide expanded datasets for testing hypotheses of genomic regulation and could potentially accelerate the development of new drugs/therapies through computational design of DNA sequences with desired biological functions.

Beyond generation evaluation, some recent gLMs demonstrate impressive zero-shot performance on predicting the effects of non-coding variants, of which the best performing ones include GPN-MSA^9^ and Evo2^1^. Clinically, this capability could integrate with existing genomic testing pipelines, flagging potentially pathogenic regulatory variants that current screening methods miss, particularly for complex or rare disorders with known genetic components.

## Ethical considerations related to the development and clinical implementation of gLMs

Beyond these technical challenges, Evo2 raises important questions about the responsible implementation of gLMs. The translation of gLMs from research to clinical will happen after these models at least capture known and non-trivial genomic signals (beyond dinucleotide frequencies), can help formulate novel hypotheses about genomic function, produce sequences with lab-validated biological functions, and establish robust performance across diverse genomic contexts and populations. As these models approach practical application, ethical considerations become increasingly important.

Evo2’s development involved scaling challenges beyond computational resources, including careful decisions about which genomic data to include in the training set. To reduce potential misuse, the authors excluded viral genomes that infect eukaryotic hosts, aiming to prevent the generation of harmful infectious agents. This risk management and assessment was achieved through collaboration with multidisciplinary experts across health security centers, public health and law schools, and medicine, health policy, and biomedical data science departments at major academic institutions^16^, setting an important precedent for the field. However, despite removing viral genomes from the training set, malicious fine-tuning could easily circumvent this safety measure by adapting the model to design such genomes with minimal additional data and compute^17^.

New ethical concerns emerge as gLMs advance, particularly around privacy and consent, dual-use risks, and access/equity. Currently, the 128,000+ genomes Evo2 trained on are open-source, but once gLMs can accurately detect clinically relevant DNA variants, they are likely to be applied in clinical settings on individual human DNA. In that case, these models will need to be implemented in such a way that individuals can consent to whole-genome variant-risk screening and maintain privacy over both their genetic data and the predictions gLMs make on patient DNA. Additionally, as these models advance in their ability to generate whole genomes and potentially new organisms, we must consider dual-use scenarios where legitimate research tools could be repurposed for harmful applications like designing new viral infections as biological weapons. Furthermore, because it is so difficult to understand how and what these models learn, misuse of these models due to ignorance is just as, if not more dangerous, than repurposing them with malicious intent^18^. Finally, implementing whole-genome sequencing for entire populations and implementing gLMs to predict on these genomes will be expensive, due to the size of large gLMs and the costs of running predictions on them alone. Integrating gLMs into existing medical systems, therefore, may have cost barriers. This could create healthcare systems where advanced, accurate genomic prediction is available only to higher-income populations, thereby exacerbating health inequities. Therefore, it would be prudent to consider AI-based regulatory frameworks, such as the one described by Derraz and colleagues (2024) in precision oncology, prioritizing human oversight, patient-centeredness, and comprehensive risk assessments in the development/implementation of gLMs^19^. Before deployment, principles of AI safety, data privacy, and equity should guide the safe and ethical development of gLMs^20^.

## Conclusions

The future of gLMs is both promising and uncertain. While they could transform medicine by decoding the genome’s regulatory mechanisms, their full impact has yet to be realized. Most of the current evaluation strategies for gLMs fail to differentiate whether their predictive capabilities are the result of true genome comprehension or statistical recapitulation of training sequences. However, gLMs’ current distance from clinical deployment may be an opportunity, allowing time to establish strategies for their safe and effective application to improve human health.

## Data Availability

No datasets were generated or analyzed during the current study.
